# Willingness to pay for National Health Insurance Fund among public servants in Juba City, South Sudan: a contingent evaluation

**DOI:** 10.1186/s12939-017-0650-7

**Published:** 2017-08-30

**Authors:** Robert Basaza, Paul Kon Alier, Peter Kirabira, David Ogubi, Richard Lino Loro Lako

**Affiliations:** 1 Institute of Public Health and Management, International Health Sciences University, Kampala, Uganda; 2 Directorate of Policy, Planning, Research and Budgeting, Ministry of Health, Juba, Republic of South Sudan

**Keywords:** National health insurance fund, Willingness to pay, Universal health coverage, South Sudan

## Abstract

**Background:**

This study assessed willingness to pay for National Health Insurance Fund (NHIF) among public servants in Juba City. NHIF is the proposed health insurance scheme for South Sudan and aims at achieving universal health coverage for the entire nation’s population. One compounding issue is that over the years, governments’ spending on healthcare has been decreasing from 8.4% of national budget in 2007 to only 2.2% in 2012.

**Methods:**

A cross-sectional study design using contingent evaluation was employed; data on willingness to pay was collected from 381 randomly selected respondents and 13 purposively selected key informants working for the national, state and Juba County in September 2015. Qualitative data were analysed using conceptual content analysis. T-tests and linear regressions were performed to determine association between WTP for NHIF and independent variables.

**Results:**

Up to 381 public servants were interviewed, of which 68% indicated willingness to pay varying percentages of total monthly individual income for NHIF. Over two-thirds (67.8%) of those willing to pay could pay up to 5% of their total monthly income, 22.9% could pay up to 10% and the rest could pay 25%. Over 80% were willing to pay up to 50 SSP (1 USD = 10 SSP) premiums for medical consultation, laboratory services and drugs. The main factors influencing the respondents’ decisions were awareness, alternative sources of income, household size, insurance cover and religion.

**Conclusions:**

Willingness to pay is mainly influenced by awareness, alternative sources of individual income, household size, insurance cover and religion. Most of the public servants were aware of and willing to pay for NHIF and prefer a premium of up to 5% of total monthly income. There is need to create awareness and reach out to those who do not know about the scheme in addition to a detailed analysis of other stakeholders. Consideration could be made by the Government of South Sudan to start the scheme at the earliest opportunity since the majority of the respondents were willing to contribute towards it.

## Background

Health insurance is a way of pooling resources so that risks are shared among different willing individuals [1]. Its most important concepts are risk pooling and sharing [2]. Some countries use a mix of social health insurance-the Bismarck Model, private and community-based schemes to afford their citizen’s social health protection (SHP) [3]. Globally, the mean willingness to pay (WTP) for health insurance among the lower and middle-income countries is estimated at 1.18% of GDP per capita and 1.39% of adjusted net National income per capita [4]. In Africa, SHP is considered an old concept; values such as the “brother’s keeper” based on solidarity within the family, clan and community were well-known in traditional African societies [5].

In South Sudan, health services at government health facilities are entirely free [6]. However, it is a low-income country [7] and majority (83%) of the estimated 11.9 million people live in rural areas with no or minimal access to healthcare facilities due to fewer numbers of health facilities and inaccessibility [8]. This has resulted in some of the worst health indicators in the world; infant and under-five mortality rates are reportedly at 102 and 135 per 1000 live births respectively [6]. Maternal mortality stands at 2054 per 100,000 [9], malaria kills an estimated 44,000 annually [8, 10] while the overall life expectancy at birth is 42 years. The fertility rate is among the highest in the world at 6.7% children per woman with 90% of women delivering without the help of skilled health professional [9]. These are worse than the sub-Saharan African average indicators where infant and under-five mortality rates are 60.8 and 91.2 per 1000 live births, respectively, life expectancy at birth is 58.5 and fertility rate is 5.1 children per woman [11].

### Health financing in South Sudan

One compounding issue is that over the years, government spending on healthcare has been decreasing from 8.4% of the national budget in 2007 to only 2.2% in 2012 [8]. The fiscal year 2015/2016 budget allocation for healthcare was 317 million SSP (3%). The national health accounts indicate that 2014 spending on health care was 2.2% of GDP, while per capita allocation was 52 USD [12]. Government spending as a percentage of total healthcare expenditure (THE) was reported at 38.8% while it was estimated at 2.6% of GDP in 2012 [13]. More than half (61.3%) of THE comes from private sources, of which 92.5% are directly out-of-pocket [13]. Private health insurance is very low and currently covers only 5.4% of the population [12]. The rest of healthcare services, especially, primary health care (PHC), is financed through NGOs [8].

“There was a previous attempt by then unified Republic of Sudan to put up a national Health insurance scheme. The South Sudan government Ministry of Gender, Child and Social Welfare Policy of 2013 points out that NHIF Act was enacted in the Sudan in 1994 and extended to Southern Sudan in 2001. The Act was further amended in 2003 to ensure stability in funding the system in order to achieve equity objectives regardless of ability to pay. This was a time of civil war and it could not succeed because of extreme poverty of the population and limited political will by then government. People could not afford the premiums and the government concentrated on the war. In addition, five of the ten states were left out in the Southern region and the nationwide equity and solidarity objectives could not be realized”.

In Juba City, healthcare funding is a joint effort of the national, state and local governments and private sources comprising the NGO’s, faith-based private not for profit entities and private for-profit healthcare. The city houses Juba Teaching Hospital, (the only national referral hospital), the Juba Military Referral Hospital, the Police Hospital, Alsabbah Children’s Hospital which is run by Central Equatoria State (CES) and primary health units and centres run by Juba County. There are also 89 private clinics, 28 medium-sized medical centres and hospitals as well as 138 drug shops, 75 pharmacies and 105 pharmaceutical companies [14]. The Juba Teaching Hospital is financed and managed by the national MOH, Alsabbah Children’s Hospital by CES while the organised forces run their health facilities [15].

This research examined the willingness to pay for the National Health Insurance Fund (NHIF) among public servants in the city. The National Health Insurance Fund is a proposed health financing mechanism in the Republic of South Sudan. Such information is crucial at such moments when the scheme is still in its early planning stages.

### Objectives

The main objective was to assess willingness to pay for national health insurance fund among public servants in Juba city, South Sudan. Specifically, we looked at the level of awareness about NHIF among public servants, the factors that influence their willingness to pay for NHIF, as well as the premium public servants are willing to pay for NHIF in Juba City.

## Methods

A cross-sectional study design using contingent evaluation was employed to gather primary data on willingness to pay from 381 randomly selected respondents and 13 purposively selected key informants working for the national, state and Juba County governments in September 2015. Modified Kish and Leslie Formula (1965): n = $\frac{Z^{{}^2} PQ}{{\mathrm{D}}^{{}^2}}$ for a known population was used to estimate the sample size [16]. In this formula: n is the sample size, Z is the confidence level (95%), P is the estimated proportion with the attribute that is in the population = 0.5 (this is because NHIF is not yet fully functional at the national level);Q is the complement of P (Q = 1-P) = 0.5 and D is the maximum error of 0.05 (Israel, 1992). The obtained sample size was further adjusted to cater for an estimated 10% non-response rate. Stratified random simple procedure was used to estimate the sample sizes of the national (98,764), state (8,000) and local government (4,000) employees making a total of 110,764. Samples of 376, of 31 and 15 respondents were drawn from the national, state and local government employees respectively making up a total sample of 422. Ministries and Departments considered in the study were randomly selected. Only employees below the rank of a Director General were given questionnaires provided they consented to participate in the study. A response rate of 90% (381 respondents) was achieved. Senior officials above the rank of Director were included in the key informant interviews after purposive selection. Uniformed employees were also excluded because they have different health care arrangements.

Willingness to pay for NHIF was the dependent variable in this study; it was the factor being influenced by other factors: socioeconomic factors (age, sex, marital status, religion, health status and family size, education, individual income level, alternative source of income), occupational factors (workstation, nature of work, work-related injuries), health system factors (distance to health facilities, quality and availability of health services) and NHIF factors (knowledge, packages offered and private insurance).

Qualitative data were analysed using conceptual content analysis and reported in quotes in appropriate sections of the results while quantitative data were entered using Epidata version 3.1 and analysed using SPSS version 16.0. T-tests and linear regression were performed to determine the association between WTP for NHIF and independent variables. Quality was maintained by pretesting questionnaires with 15 respondents, training and selecting Research Assistants from those who had experience in data collection and fluent in written and spoken English as well as working knowledge of Arabic. One of the authors (KPA), carried close supervision of the entire process of data collection in order to respond to queries also ensured that the process maintained quality.

“For confidentiality, respondents’ names of were not written on the questionnaires and were assured of concealment of the information provided. This was kept throughout the process. The informed consent was sought from all authorities and each individual before data collection. One of the authors (KPA) supervised the data collectors throughout the entire data period. Every respondent filled out the questionnaire without influence from colleagues, bosses nor data collectors”.

## Results

### Socio-demographic characteristics of respondents

Socio-demographic characteristics of the respondents are presented in Table 1. Most of the respondents (35%) were in the age group 28–37 years, and 27% in the youngest age group, 18–27 years. Minority respondents (7%) were found to be 58 or more years old. It was also found that most of the respondents (71%) were males compared to 29% females. Sixty-five percent (65%) said they were in monogamous marriages followed by polygamous ones (17%). The study found divorce or separated cases to be the least at only 2%. “The analysis of data was along household sizes: 1-4, 5-8, more than 8 is in line with the East African Social health Protection Report of 2014 that provides for this categorization in the upcoming and launched NHIS schemes (South Sudan is the newest member country of East African Community). The schemes therein the report provide social health protection for up to 4 family members and extra contribution subject to family sizes of 5-8 and further lower rate or no charge for those of 8 or more people,”.The modal household size (37%) consisted of 1–4 members and this was closely followed by households of 5–8 members (36%) and the rest of family sizes form the remaining percentage. While most of the respondents (87%) were found to be Christians, 12% were Muslims and just 0.5% said they believed in African Traditional Religions. On health status, most of the respondents (86%) did not experience any chronic health condition as compared to those who had chronic ailments (14%).

**Table 1:** Socio-demographic characteristics of respondents (*N* = 381)

Variable	Descriptions	Frequency	Percentage (%)
Age of respondents	18–27	98	(26.72%)
	28–37	135	(35.43%)
	38–47	81	(21.26%)
	48–57	39	(10.24%)
	58 and above	28	(7.35%)
Sex of respondents	Male	271	(71.13%)
	Female	110	(28.87%)
Marital status	Monogamous	248	(65.09%)
	Polygamous	64	(16.80%)
	Separated or divorced	6	(1.57%)
	Widowed	8	(2.10%)
	Single	55	(14.44%)
Household size	1–4	139	(36.48%)
	5–8	137	(35.96%)
	9 and above	105	(27.56%)
Religion	Christianity	332	(87.14%)
	Islam	47	(12.34%)
	African traditional belief	2	(0.52%)
Health status (chronic illness)	Yes	55	(14.44%)
	No	326	(85.56%)
Education level	Tertiary level	194	(50.91%)
	Secondary	136	(35.70%)
	Primary	42	(11.02%)
	No formal education	9	(2.36%)
Private insurance	Yes	62	(16.27%)
	No	319	(83.73%)
Monthly salary (SSP)	1–1000	191	(50.13%)
	1001–2000	111	(29.13%)
	2001–3000	58	(15.22%)
	3001–4000	12	(3.15%)
	4000 and above	9	(2.36%)
Other sources of income	Yes	106	(27.82%)
	No	275	(72.18%)
Total monthly income (SSP)	1–1000	151	(39.63%)
	1.001–2000	118	(30.97%)
	2001–3000	51	(13.39%)
	3001–4000	28	(7.35%)
	4000 and above	33	(8.66%)

(1 USD = 10 SSP at the time of this research); Source: Data collection by Research Team

Four education levels: tertiary (any studies above secondary education), secondary (respondents holding a South Sudan Certificate of Secondary Education that is 11 years of education or equivalent), primary (responding holding a primary leaving certificate that is 7 years of education or equivalent) and no formal education (those who do not fall in the above categories) were elicited from the respondents. They were also asked whether they had insurance coverage, what range their monthly salary was, whether they had other sources of income and to state their total monthly income. More than half of respondents (51%) had reached post-secondary levels while only 2% had no formal education.

Over a third (36%) had at least completed secondary school while 11% reached primary level only. More than four-fifths of the respondents (84%) did not have any insurance coverage while half (50%) said they earned a monthly salary between 1 and 1000 South Sudanese pounds (SSP). Close to a third (29%) earned between 1001 and 2000 SSP while only 9 (2%) said they earned more than 4000 SSP. Close to three quarters (72%) had no other sources of income outside the government pay whereas close to a third (28%) of respondents reported having other sources of income that supplemented their government pay.

### Ownership of household assets and access to social amenities

A good measure of economic status is ownership of household assets and access to social amenities. “The study measured the proportion of respondents owning each household item.” (Table 2); 90% of respondents owned a mobile phone followed by a television set (48%), computer (37%) and electricity (32%). The least owned items were microwave oven and fixed telephone both being owned by just 2% of the respondents.

**Table 2:** Ownership and access to household assets and amenities (*N* = 381)

Household assets	Yes	No
Electricity	120 (32%)	261 (69%)
Running water (tap)	39 (10%)	342 (90%)
Water closet toilet	65 (17%)	316 (83%)
Solar power	57 (15%)	324 (85%)
Television	181 (48%)	200 (53%)
Vehicle	95 (25%)	286 (75%)
Fridge	81 (22%)	300 (78%)
Indoor bathroom	93 (24%)	288 (76%)
Laundry machine	12 (3%)	369 (97%)
Computer	142 (37%)	239 (62%)
Micro wave oven	6 (2%)	375 (98%)
Fixed telephone	6 (2%)	375 (98%)
Mobile telephone	341 (90%)	40 (10%)

Source: Data collection by Research Team

### Annual expenditure on selected basic needs

In an effort to determine their spending behavior, respondents were given a list of selected basic needs such as communication, education, food and transport and were asked to indicate what took most of their earnings in a given year (Table 2). “The highest expenditure in the majority of the respondents 315 (83%) was on food, followed by 40 (11%) on education and 20 (5%) on healthcare”. The remaining 1% comprised of: 3 respondents who said they spend most of the income on communication just like another group of 3 who indicated their highest expenditure on transport. Despite almost all respondents reporting having mobile phones, communication did not feature most in the list of expenditures.

### Distance from the nearest health facility

Respondents were asked to indicate how far they lived from the nearest health facility; most of the respondents (65.6%) in Juba City indicated they live within or 2 km, 80 (21%) live more than 2 and within or 6 km while the rest (51) live more than 6 km from the nearest health facility.

### Preferred health facility

**Fig. 1:**
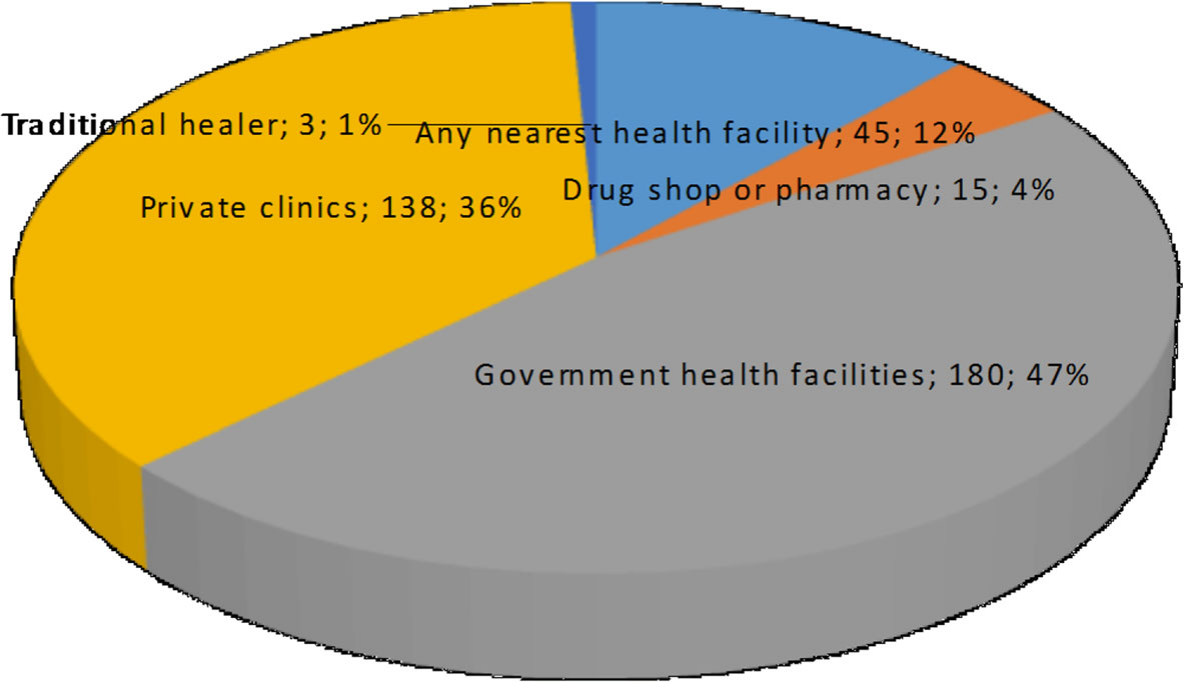
Preferred health facility

Similarly, respondents were asked to choose which health facility they preferred when they or members of their families felt sick (Fig. 1). Out of 381 respondents, 47% would seek healthcare at government health centres while 36% preferred private clinics. Over a tenth (12%) would go to any the nearest facility while 4% preferred drug shops or pharmacies. The least preferred place of treatment was traditional healers.

### Reasons for the choice of health facility

Some of the reasons the respondents advanced for the choice of health facility included proximity as expressed by those whose choice was any nearest health facility. Those who preferred government facilities cited affordability of treatment, trust and free services as some of their reasons. Respondents who preferred private clinics mentioned quick and timely services, accurate or proper diagnosis, quality care and availability of drugs as the determinants of their choices.

### Occupational injury status

Respondents were also asked whether they ever had injury at their places of work and out of 381 respondents interviewed, most (85.2%) did not experience any occupational injury. Only a small proportion of the respondents (14.8%) mentioned having had injury at the workplace.

### Awareness about NHIF

Respondents were asked whether they were aware of the proposed NHIF and slightly over a half of respondents (52.8%) indicated that they knew about the proposed plan while the rest (47.2%) knew nothing about it. Some respondents were able to define NHIF well as one respondent articulated “*Monthly contributions by the working class to support themselves and their families during ill-health*” (KI, Health worker Alsabbah Children’s hospital), and another termed it as “*part of cost sharing*” since “*government alone cannot satisfy all health needs due to limited funds*” (KI, Staff of Drug and Food Control Authority headquarters). Those who did not know about it wondered where it was; one respondent emphatically put it, “*I do not know about it, where is it (the draft proposal)? It should be distributed to all ministries. I only knew about the NHIF of the Sudan*” (KI, staff of Central Equatoria State Ministry of Public Service headquarters).

### Premium

Respondents were asked whether they were willing to pay (WTP) for NHIF if it were introduced and over two-thirds (68%) expressed WTP for the scheme and only 32% were not interested. Out of 258 respondents who expressed WTP for NHIF, 175 (67.8%) favored paying up to 5% of their income, 22.9% would pay between 6 and 10% while the rest (9.3%) could pay a premium of over 10%. Cumulatively, over 90% were willing to pay up to 10% of their income. Across the five income groups, percentage contribution remained fairly constant because most of the respondents in all the income groups indicated WTP up to 5% of their total monthly income for NHIF. It is only in the fifth income grouping that more than half indicated they could pay higher percentages but still most of these settled for up to 10% of total income (Table 3).

**Table 3:** Ownership and access to household assets and amenities (*N* = 381)

Household assets	Yes	No
Electricity	120 (32%)	261 (69%)
Running water (tap)	39 (10%)	342 (90%)
Water closet toilet	65 (17%)	316 (83%)
Solar power	57 (15%)	324 (85%)
Television	181 (48%)	200 (53%)
Vehicle	95 (25%)	286 (75%)
Fridge	81 (22%)	300 (78%)
Indoor bathroom	93 (24%)	288 (76%)
Laundry machine	12 (3%)	369 (97%)
Computer	142 (37%)	239 (62%)
Micro wave oven	6 (2%)	375 (98%)
Fixed telephone	6 (2%)	375 (98%)
Mobile telephone	341 (90%)	40 (10%)

Source: Data collection by Research Team

The idea of making contributions as percentages was applauded because it would make people pay according to ability.

“*I have not seen the draft proposal but the idea of percentage is to enable people to pay according to capacity…*” (KI, Staff of Drug and Food Control Authority Headquarters)

### Respondents’ WTP for proposed health care services

A list of proposed NHIF healthcare services was presented to the respondents; they were to indicate whether to pay or not for each of these. More than half of the respondents (59.6%) were willing to pay for medical consultation followed by laboratory services (44.9%) and drugs (36.5%). Over a quarter (25.2%) of the respondents was willing to pay for maternal health services while (21.8%) said they would pay for x-ray and other radiological procedures. The least favored health service was health promotion with only 17% willing to pay. Asked how much they would pay for each service, more than 80% of respondents in each case suggested WTP to up to 50 SSP for each of the suggested service packages.

The reasons given for willing to pay for NHIF included perception of the fund by public servants as part of their welfare scheme which would take care of their healthcare and that of their families when sickness strikes. They also believed it would bring down the cost of healthcare in addition to improvement of its infrastructure. Some even went as far as suggesting that this program would reduce poverty levels in the country.

“*By being a member of health insurance, I will benefit by sharing the amount of money which may be needed from me for healthcare services from what I can afford*” (KI, Staff of MOH headquarters)

Another reason was given uncertainty of disease occurrence. Paying for NHIF was seen as providing social security because a disease does not alert people when it would strike.

“*Sickness does not inform you that I am coming. That is why it is good to contribute. Imagine getting sick and nowhere to borrow just like in the current economic crisis, what will happen? It is good to contribute even if one does not fall sick…*” (KI, Health worker Alsabbah Children’s Hospital).

It was observed that paying for the scheme would make people value health care services and thereby improve utilization.

“*When people contribute to service, they attach value to such services and access becomes easier*” (KI, staff of Drug and Control Authority HQs).

On the other hand, those who were not willing to pay for NHIF cited inadequate information on the scheme, limited or unreliable income, corruption and mistrust and prior bad experience with the former NHIF of the Republic of the Sudan, where services did not meet the contributors’ expectations.

“*I am not aware of the benefits*” (KI, staff of Juba County HQs)

The results of the respondents’ willingness to pay and ownership of assets are presented in Table 4. The assets considered for this study were: solar lighting system, a television set, a vehicle, a fridge, indoor bathroom, laundry machine, personal computer and microwave. This is a select list from the South Sudan Household Health Survey 2010. To get the number of assets a given respondent had, we summed the household assets from 0 to 8, with possession of assets =1 and no assets =2, thus a total of all assets = 8. Table 4 (a) shows that close to two thirds (62%, 79/259) with no assets are willing to pay and 100% of those with assets (7–8 category) are willing to pay. Table 4(b) shows that the close to two thirds of the majority (62.2%, 79/127) of those who don’t possess assets are willing to pay compared to the 37.8% of those who are not willing to pay. Over seven tenth (70.9%, 180/254) of those with assets are willing to pay compared to the 29.1% who possess assets but are not willing to pay. Table 4(c) shows that there was no significant relationship between willingness to pay and the possession of assets (*P* = 0.088 > 0.05). In addition, a unit change in the possession of assets affected the WTP by 0.39 points. Those who possess assets are 1.48 times more likely to pay for insurance than those who don’t possess assets. With the Wald statistic 2.903 and 1df, we fail to reject the null hypothesis and conclude that there is no significant relationship between possession of assets and WTP.

**Table 4:** Respondents willingness to pay and possession of individual assets

a) Willing to pay against the number of assets											
		Number of assets									Total
		0	1	2	3	4	5	6	7	8	
Willing to pay		79	67	30	28	25	23	4	2	1	259
		62%	76%	61%	72%	64%	77%	67%	100%	100%	68%
Not willing to pay		48	21	19	11	14	7	2	0	0	122
		38%	24%	39%	28%	36%	23%	33%	0%	0%	32%
Total		127	88	49	39	39	30	6	2	1	381
b) Willing to pay against the number of assets											
		Possession of assets									Total
		Doesn’t possess assets						Possess assets			
Willing to pay		Count			79			180			259
		% within			62.2%			70.9%			68.0%
Not Willing to pay		Count			48			74			122
		% within			37.8%			29.1%			32.0%
Total		Count			127			254			381
		% within			100.0%			100.0%			100.0%
c) Binary logistic regression											
Variables in the equation											
		B	S.E.		Wald	df			Sig.		Exp (B)
Step 1^a^	PA (1)	.391	.229		2.903	1			.088		1.478
	Constant	−.889	.138		41.435	1			.000		.411

Source: Data Collection by Research Team

*PA* Possession of assets

^a^Variable (s) entered on step 1: PA

## Discussion

### Factors influencing WTP for NHIF in Juba City

The high number of respondents (85.2%) who did not experience any occupational injury was due to the majority who worked in their offices performing duties which largely involves documentation.

Despite the fact that some of those who expressed unwillingness to pay for NHIF cited low income as the major stumbling block for their reservation, total monthly income was not a significant determinant of WTP for NHIF. This was a surprising finding because elsewhere it was observed in a study on WTP for community-based health insurance in Nigeria that the poorest indicated the lowest WTP of 193 Naira compared with the least poor who suggested a WTP of 329 Naira (the average Naira: USD exchange in 2012 was 157: 1) [17]. On the effect of age and frequency of falling sick on WTP, Oyekale (2012) found a strong negative correlation [18] but Babatunde et al. found a significant influence (*p* = 0.000) of age on WTP for health insurance [19]. Our findings are in agreement with Oyekale but inconsistent with Babatunde et al.; age and chronic illness were found to be insignificant. However, most of the respondents (61.1%) in this study were between 18 and 37 years old unlike in the Oyekale case where those less than 40 years old constituted only 27.9% of the respondents, the bulk being senior citizens (over 60 years old) who probably experienced an array of illnesses. This suggests there are more intervening factors in determining WTP other than age. While this study found household size as a significant determinant (*p* = 0.049) of WTP, Oyekale (2012) found a negative correlation (−0.1756366) just like what Dror et al. (2006) found in their study on WTP for health insurance among rural and poor persons in India [20]. Dror et al. concluded that household composition did not affect WTP.

This study found that awareness is a significant influence on WTP for NHIF. This is consistent with the findings of Oyekale who also found that WTP for CBHIS increased significantly with awareness. A similar finding was also reported by Biosca & Brown (2014) in their study on boosting health insurance in developing countries and stressing the effect on conditional transfers in Mexico [21]. However, this was not the case in a study carried out by Bawa and Ruchita in Punjab India where 71% of the respondents reported being aware but were not subscribed to health insurance [22].

### Awareness of public servants in juba about NHIF

The research found that more than half of the public servants in Juba City, who participated in the study, were aware of NHIF while the rest did not know anything about it. This could be explained by the fact that “South Sudanese civil service is made up of mainly two groups of people: those who served in the Sudanese Civil Service and knew about NHIF and those who were employed after independence and knew nothing about the scheme before”. Oyekale (2012), found less than half (49.1%) of respondents aware of CBHIS in Nigeria possibly because his study population was mainly rural [18]. In Mexico, Biosca & Brown (2014) found that awareness remains a vital boost to health insurance in developing countries [21]. It can thus be predicted that NHIF stands to gain from a high percentage awareness.

### Premium

In social health insurance schemes, premiums are mostly charged as percentages of income. This is one of the most important principles of health insurance [1] because it ensures that contributions are according to ability while access to services is based on need. It is apparent from this study that over two-thirds of public servants in Juba City are willing to pay for NHIF: 45.9% of these are willing to pay a premium of up to 5% of their total monthly income, 15.5% indicated they could pay up to 10% and the rest (38.6%) indicated higher percentages. This premium is higher than what was found among the informal sector in China [23] possibly because this study mainly considered salaried workers unlike the Chinese study which focused on low-income earners. Dror et al. (2006) study in rural India even found a much lower premium further elaborating the level of poverty in these communities. This premium is also consistent with the proposed 4% contribution by employees for Uganda’s proposed National Health Insurance Scheme [5]**.**

### Study limitations

There was limited understanding of the value of this study among some senior government officials and could not readily provide access to their subordinates. Some went as far as calling it a private affair but were convinced by their peers and consented. In addition, absence of some of the public servants due to missions outside the city and abroad among other reasons could also have impacted on the sampling procedure. As much as possible, the research team kept to the sampling procedures and had to wait for the officials to return from abroad.

The results from this study are not generalizable across the entire public servants in South Sudan. They are only specific to Juba City. They do provide lessons to the entire public service in Juba and the rest of the country. In order to generalize for the whole population, there is need to include the other population of public servant outside Juba City, uniformed services, the formal and informal business sectors and the rural population in a further study. It is then that equity objectives may be inferred. Uniformed services are estimated to be three hundred thousand (2.5% of the entire population) and consist of the army, the police, prison, wildlife, civil defence force and the national security services. In some countries, these groups have different health insurance packages. In South Sudan, National Security services are covered by the State while the rest benefit from outright allocations from the government.

## Conclusions

The findings from this study provide an insight into the factors that will likely influence public servants’ willingness to pay for the proposed national health insurance plan in Juba City, South Sudan. Some of the most prominent of these are awareness, alternative sources of income, household size, insurance coverage, ownership of household assets and religion.

More than two-thirds of public servants in Juba City are willing to pay for national health insurance fund. Of these, a majority is ready to pay 5% or less of their total monthly income to the scheme and up to 50 SSP (2.4 USD) for each of the services received from health facilities. This is relatively uniform across all income groups. They prefer to pay for medical consultation, laboratory services and drugs. Reasons expressed for WTP included perceived benefits like cost sharing, development of healthcare infrastructure, risk protection, and reduction of poverty. Reasons for not willing to pay included other insurance coverage, low income, corruption and mistrust as well as inadequate information available about the scheme.

The preferences of the prospective premium are within what has been suggested in the draft proposal on the establishment of the fund in the country. It is possible that the scheme may be well received by the public servants in Juba City once it is introduced.

The study recommends:

- The Government of South Sudan could run a public campaign and provide more information on the national health insurance scheme to all stakeholders (public servants, policy makers, private sector workers and the general community) so that they own (buy-in) the program right from the start. Some public officers not willing to pay cited lack of adequate information. A task force could be established by the Ministry of Gender, Child and Social Welfare with technical support from MOH to accomplish such an undertaking. This could ensure a robust and successful start.
- The involvement of all stakeholders is crucial in fast tracking the process; it is therefore incumbent upon the national Ministry of Gender, Child and Social Welfare supported by the national Ministry of Health to engage all those who will be affected by the scheme as early as possible by carrying out adequate feasibility studies including stakeholder analysis.

## Abbreviations

CBHIS: Community Based Health Insurance Scheme
EAC: East African Community
GDP: Gross Domestic Product
HQS: Headquarters
KI: Key informant
MGC&SW: Ministry of Gender, Child and Social Welfare
MOH: Ministry of Health
NBS: National Bureau of Statistics
NGO: Nongovernmental organisation
NHIF: National health insurance fund
PHC: Primary Healthcare
SHI: Social Health Insurance
SHP: Social Health Protection
SPLA: Sudan People’s Liberation Army
SPSS: Statistical package for social sciences
SSP: South Sudanese pound
TH”: Teaching Hospital
THE: Total health expenditure
UHC: Universal health coverage
USD: United States dollar
WHO: World Health Organisation
WTP: Willingness To Pay

## References

[1] Kutzin J (1997). Health Insurance for the Formal Sector in Africa: yes, But.

[2] Okuonzi SA (2009). Free-market illusion: health sector reforms in Uganda 1987–2007.

[3] Witter S, Ensor T, Jowett M, Thomson R (2000). Health economics for developing countries: a practical guide.

[4] Nosratnejad S, Rashidian A, Dror DM (2016). Systematic review of willingness to pay for health Insurance in low and Middle-Income Countries. PLoS One.

[5] East African Community (2014). Situational analysis and feasibility study of options for harmonisation of social health protection options for universal health coverage in east African community partner states.

[6] Ministry of Health. *Health Sector Development Plan* 2012–2016. Juba: Ministry of Health. p. 2012.

[7] World Bank. The World Bank*.* 2015 Available at: http://www.worldbank.org. Accessed 20 Oct 2015.

[8] Downie R (2012). State of public health in South Sudan:critical condition.

[9] Oxfam (2013). Country profile: South Sudan.

[10] National Bureau of Statistics. National Bureau of Statistics. 2010. Available at: http://www.ssnbss.org/. Accessed 9 Oct 2015.

[11] United Nations Development Program (2015). Human development report 2015: work for human development.

[12] Knoema. World Data Atlas*.* 2013 Available at: http://guineab.opendataforafrica.org. Accessed 21 Mar 2016.

[13] Taggart R M. Quandl*.* 2015. Available at: https://www.quandl.com/collections/south-sudan/south-sudan-health-data. Accessed 10 Oct 2015.

[14] Ministry of Health, Drug and food control authority and Jubek state Ministry of Health*:* South Sudan Ministry of Health. 2001.

[15] Ministry of Health. Basic package of healthcare and nutrition Services for Southern Sudan. Juba: South Sudan Ministry of Health, 2009.

[16] Israel GD (1992). Determining sample size.

[17] Obinna O, Ekechi O, Chima O, Benjamin U, Joses K, Petu Amos O (2010). Willingness to pay for community-based health insurance in Nigeria: do economic status and place of residence matter?. Health Policy Planning.

[18] Oyekale AS (2012). Factors influencing Households' willingness to pay for National Health Insurance Scheme (NHIS) in Osun state, Nigeria. Ethno Med.

[19] Babatunde OA, Akande TM, Salaudeen AG, Aderibigbe SA, Elegbede OE, Ayodele LM (2012). Willingness to Pay for Community Health Insurance and its Determinants among Household Heads in Rural Communities in North-Central Nigeria. International Review of Social Sciences and Humanities.

[20] Dror D M, Radermacher R, Koren R. Willingness to pay for health insurance among rural and poor persons: field evidence from seven micro health insurance units in India Health Policy. 2006; Available at. DOI: 10.1016/j.healthpol.2006.07.011. Accessed 21 Aug 2017. 10.1016/j.healthpol.2006.07.011 16971017

[21] Biosca O, Brown H (2014). Boosting Health Insurance in Developing Countries: Do Conditional Cash Transfer Programmes Matter in Mexico?. Health Policy and Planning..

[22] Bawa S K, Ruchita. Awareness and Willingness to Pay for Health Insurance: An Empirical Study with Reference to Punjab India. International Journal of Humanities and Social Science. 2011;1(7).

[23] Bärnighausen T, Liu Y, Zhang X, Sauerborn R. Willingness to pay for social health insurance among informal sector workers in Wuhan, China: a contingent valuation study. BMC Health Services Research. 2007; Volume 7. 10.1186/1472-6963-7-114 PMC2065868 17659084

